# Bilateral adrenal infarction in a patient with essential thrombocythemia: a case report and brief literature review

**DOI:** 10.1093/omcr/omaf129

**Published:** 2025-08-11

**Authors:** Yuko Fujishima, Yuji Hataya, Kanta Fujimoto, Toshio Iwakura, Naoki Matsuoka

**Affiliations:** Department of Diabetes and Endocrinology, Kobe City Medical Center General Hospital, Kobe 650-0047, Japan; Department of Diabetes and Endocrinology, Kobe City Medical Center General Hospital, Kobe 650-0047, Japan; Department of Diabetes and Endocrinology, Kobe City Medical Center General Hospital, Kobe 650-0047, Japan; Department of Diabetes and Endocrinology, Kobe City Medical Center General Hospital, Kobe 650-0047, Japan; Department of Diabetes and Endocrinology, Kobe City Medical Center General Hospital, Kobe 650-0047, Japan

**Keywords:** adrenal infarction, essential thrombocythemia, Japan

## Abstract

Adrenal infarction (AI) is rare condition caused by inadequate blood supply to the adrenal glands, with few reports of its association with essential thrombocythemia (ET). Here, we report a case of bilateral AI as an initial thrombotic complication in a patient with ET. A 69-year-old man presented with right abdominal pain. Abdominal computed tomography (CT) showed diffuse enlargement and poor enhancement of bilateral adrenal glands. He was diagnosed with bilateral AI. Based on positive screening for thrombocytosis and the Janus kinase 2 V617F mutation, he was diagnosed with ET. After low-dose aspirin therapy, he showed no symptom recurrence. The cortisol response in the adrenocorticotropic hormone stimulation test decreased at onset but improved after three months. Furthermore, abdominal CT revealed improvement in bilateral adrenal enlargement and enhancement. In patients presenting with AI, ET should be considered as a potential underlying disease, and early diagnosis and treatment is important to prevent recurrence.

## Introduction

Adrenal infarction (AI) is a rare condition characterized by the injury or necrosis of the adrenal glands due to inadequate blood supply [1]. It is commonly reported as a complication of antiphospholipid syndrome (APS), heparin-induced thrombocytopenia, and polycythemia vera. Essential thrombocythemia (ET) is a myeloproliferative neoplasm characterized by thrombotic complications, including acute myocardial infarction, peripheral arterial thrombosis, and venous thromboembolism [2]. However, there are few reports of AI associated with ET [1–5]. Herein, we report a case of bilateral AI as an initial thrombotic complication in a patient with ET and review previous reports.

**Table 1:** Laboratory findings on admission.

WBC (/μl)	10800	TP (g/dl)	7.5
Neu (%)	80	Alb (g/dl)	4.2
Lym (%)	12.5	AST (U/l)	22
Mon (%)	7.5	ALT (U/l)	21
Eos (%)	0	LDH (U/l)	297
Bas (%)	0	ALP (U/l)	207
RBC (/μl)	404×10^4^	γ-GTP (U/l)	55
Hb (g/dl)	14.1	CK (U/l)	43
Ht (%)	39.7	CRP (mg/dl)	2.51
Plt (/μl)	54.7×10^4^	BUN (mg/dl)	15.6
		Cre (mg/dl)	0.75
		Glu^*^ (mg/dl)	132
		Na (mEq/l)	136
		K (mEq/l)	4.1
		Cl (mEq/l)	102

(^*^) 3-4 h after last meal.

Case Presentation.

The patient was a 69-year-old man diagnosed with Crohn’s disease 17 years earlier. His condition was stable on 3000 mg of mesalazine without steroids. Eight days prior to visiting our hospital, he had been admitted to another hospital with left abdominal pain. The possibility of pain due to Crohn’s disease was considered, and prednisolone (40 mg/day) was administered for three days. Following treatment, his left abdominal pain improved, and he was discharged. However, right abdominal pain appeared immediately after discharge, and he visited our hospital. He had no family history of endocrine or hematological diseases and no medical history other than Crohn’s disease.

**Figure 1:**
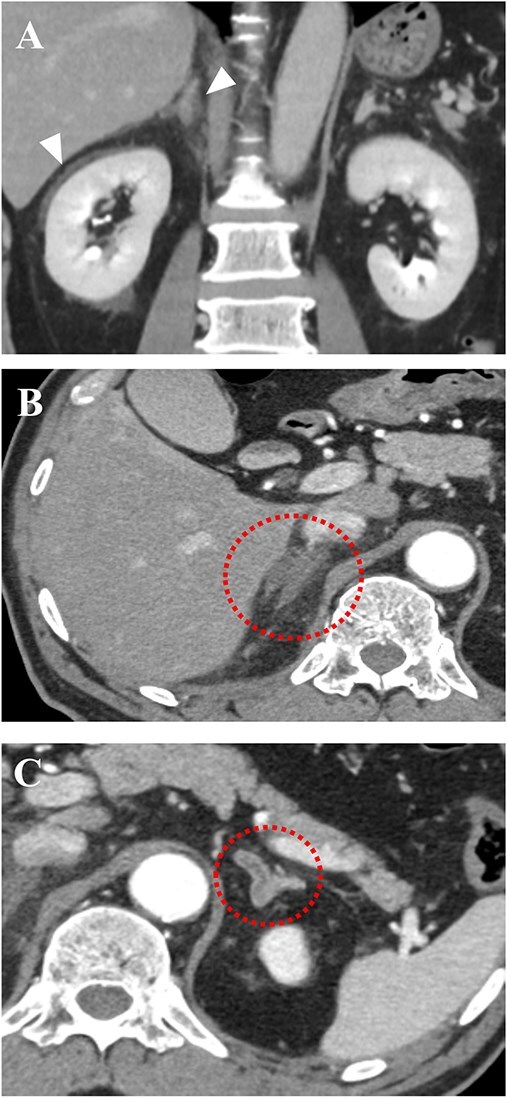
Abdominal contrast-enhanced computed tomography (CT) scans on admission. (A) Sagittal section image. Arrows indicate panniculitis around the right kidney and adrenal gland. (B and C) axial section images. Bilateral enlarged adrenal glands with no enhancement after contrast injection. On the left adrenal gland, CT scan shows no parenchymal enhancement with minimal peripheral capsular enhancement (‘capsular sign’).

On physical examination, he presented with a body temperature of 36.7°C, blood pressure of 196/104 mmHg, and pulse rate of 68 beats/min. He reported persistent right-sided abdominal pain. Resting electrocardiography and chest radiography revealed no abnormalities. Blood tests showed a mild elevation in white blood cell count (10 800/μl) and C-reactive protein level (2.51 mg/dl) (Table 1). Abdominal computed tomography (CT) demonstrated diffusely enlarged bilateral adrenal glands (Fig. 1), with poor enhancement of the right adrenal gland, absent parenchymal enhancement, and minimal peripheral capsular enhancement in the left adrenal gland. Based on these findings, he was diagnosed with bilateral AI.

**Figure 2:**
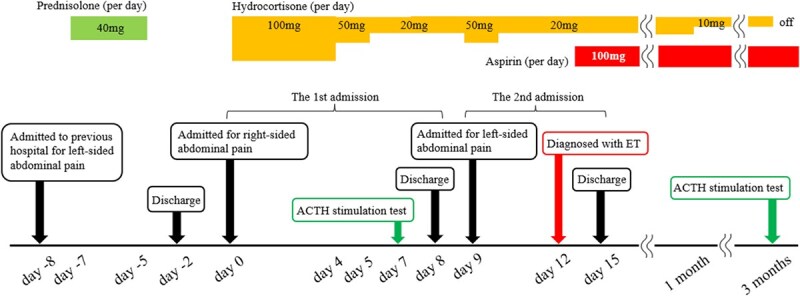
Clinical course of the patient ACTH, adrenocorticotropic hormone; ET, essential thrombocythemia.

**Figure 3:**
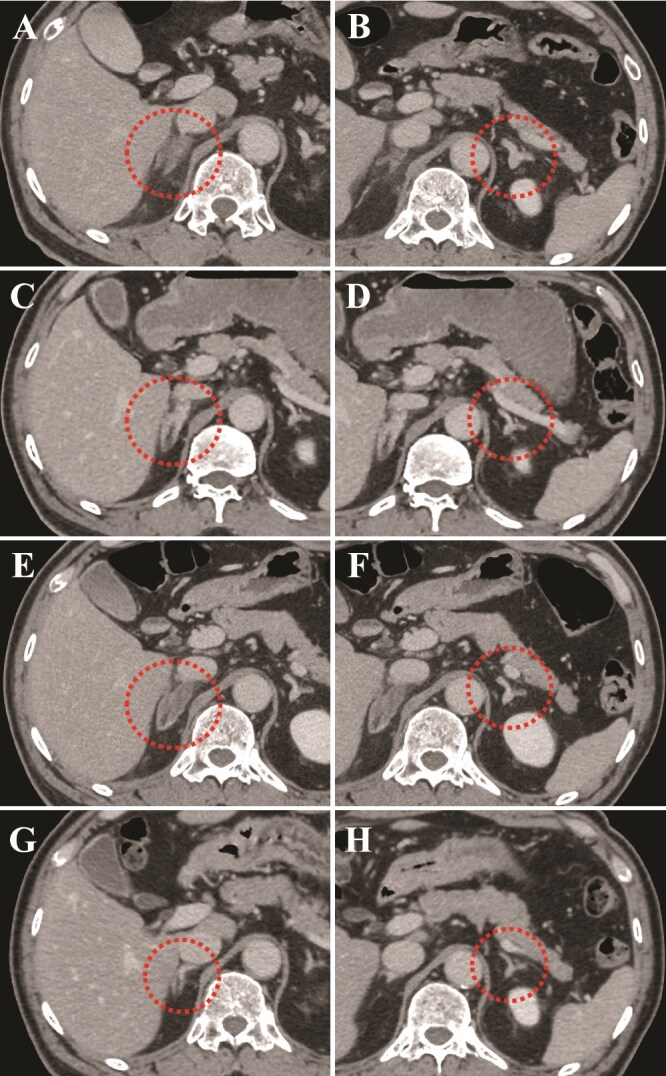
Axial section images of contrast-enhanced computed tomography scan. The left images (A, C, E, and G) show the right adrenal gland, and the right images (B, D, F, and H) show the left adrenal gland. (A and B) day 0: Bilateral enlarged adrenal glands with no enhancement after contrast injection. (C and D) day 8: Improvement in size and contrast-enhanced effect after sufficient pain control. (E and F) day 9: Bilateral enlarged adrenal glands and poor enhancement at the time of new left abdominal pain. (G and H) three months later: Bilateral adrenal glands normalized in both size and contrast-enhanced effect.

He was admitted to our hospital for conservative management due to the absence of active bleeding. A stress dose of hydrocortisone was administered, and 5 mg of amlodipine was started to manage hypertension. His clinical course and adrenal function are shown in Fig. 2 and Table 2, respectively. Following hospitalization, his blood pressure stabilized, and abdominal pain gradually diminished. Hydrocortisone was tapered, and an adrenocorticotropic hormone (ACTH) stimulation test was performed on day 7 (Table 3). Since the peak serum cortisol level was low, the hydrocortisone dose was maintained at 20 mg/day. CT on day 8 showed an improvement in the size and contrast enhancement of the bilateral adrenal glands (Fig. 3C and D). He was discharged with 20 mg of hydrocortisone; however, left-sided abdominal pain recurred on day 9. CT revealed bilateral adrenal enlargement and poor enhancement (Fig. 3E and F), leading to a diagnosis of recurrent AI and readmission. While blood tests and contrast-enhanced CT showed no findings suggestive of infection, his platelet count increased to approximately 60.0 × 10^4^/μl. A bone marrow biopsy showed slightly hyperplastic marrow with a cellularity of approximately 50%. Erythroid and myeloid lineage were also observed, but maturation was relatively normal. While megakaryocytes were increased, there was no cluster formation. Janus kinase 2 (JAK2) V617F mutation was positive; therefore, the diagnosis of ET was established based on the WHO diagnostic criteria [6]. β2 glycoprotein 1 immunoglobulin M type was initially positive but turned negative when retested three months later (Table 4), indicating that the diagnostic criteria for APS were not met [7]. Considering the high-risk group for thrombosis, low-dose aspirin was administered on day 13.

**Table 2:** Transition of adrenal function.

	Day 0	Day 1	Day 5	Day 11	Day 12	Month 1	Month 2	Month 3
Adrenocorticotropic hormone (pg/ml)	49.4	60.3	44.5	17.8		52.1	38.1	40.5
Cortisol (μg/dl)	29.9	18.0	6.0	5.2		9.8	9.8	9.9
Plasma renin activity (ng/ml/hr)		0.6			<0.2			0.5
Plasma aldosterone concentration (pg/ml)		79.8^*^			67.3^*^			78.7^**^
Dehydroepiandrosterone sulfate (μg/dl)		66.0			29.0			63.0

Plasma aldosterone concentration levels were measured using a chemiluminescent enzyme immunoassay (*: Deteminer CL Aldosterone kits, Minaris Medical Co., Ltd. Tokyo, Japan. **: Lumipulse Presto Aldosterone kits, Fuji Rebio, Co., Ltd, Tokyo, Japan). Examinations were performed in the morning except for day 0 and at least 24 h after hydrocortisone administration except for day 1.

**Table 3:** ACTH stimulation test.

Cortisol (μg/dl)	0 min	30 min	60 min
Day 7	8.0	10.9	12.0
3 months later	9.9	14.7	16.1

ACTH, adrenocorticotropic hormone.

**Table 4:** Laboratory findings regarding blood and collagen-related diseases.

Blood coagulation		(Normal range)	Collagen disease		(Normal range)
Prothrombin time (s)	13.8		Anti-nuclear antibody (number of times)	80	(<40)
Prothrombin time international normalized ratio	1.06		Rheumatoid factor (IU/ml)	4.0	(0-15)
Activated partial thromboplastin time (s)	27.6	(24.3-38.9)	Lupus anticoagulant (Normalized ratio)	0.9	(0-1.2)
Fibrinogen quantity (mg/dl)	561	(180-320)	Treponema pallidum	Negative	
D-dimer (μg/ml)	1.62	(0.00-0.99)	Rapid plasma regain	Negative	
Protein S (%)	80	(74-132)	Anti-cardiolipin immunoglobulin G type (U/ml)	3.0	(≤20.0)
Protein C (%)	118	(64-135)	Anti-cardiolipin immunoglobulin M type (U/ml)	5.1	(≤20.0)
Antithrombin III (%)	85.2	(70.0-130.0)	β2 Glycoprotein 1 immunoglobulin G type (U/ml)	<6.4	(≤20.0)
			β2 Glycoprotein 1 immunoglobulin M type (U/ml)	30.8	(≤20.0)
Genetic mutation					
Janus kinase 2 V617F	Positive		3 months later		
Janus kinase 2 exon12	Negative		Anti-cardiolipin immunoglobulin G type (U/ml)	<2.6	(≤20.0)
Myeloproliferative leukemia	Negative		Anti-cardiolipin immunoglobulin M type (U/ml)	6.7	(≤20.0)
Calreticulin	Negative		β2 Glycoprotein 1 immunoglobulin G type (U/ml)	7.7	(≤20.0)
			β2 Glycoprotein 1 immunoglobulin M type (U/ml)	17.6	(≤20.0)
Bone marrow biopsy findings					
Cellularity	~50%				
Cluster formation	Negative				
G-banding	46, XY, +1, der(1;7)(q10;p10)				

Three months later, CT revealed improvement in the bilateral adrenal glands (Fig. 3G and H). The hydrocortisone dose was reduced to 10 mg/day, and the ACTH stimulation test was repeated. His cortisol level after ACTH stimulation improved (Table 3), allowing for the discontinuation of hydrocortisone treatment. Low-dose aspirin was continued, and his clinical condition was stable with no symptom recurrence after one year.

## Discussion

The JAK2 V617F mutation is present in approximately half of ET cases, and recent reports suggest that patients harboring this mutation experience a higher incidence of venous thrombosis [8]. According to the International Prognostic Score for ET (IPSET), patients over 60 years of age with this mutation are classified as a high-risk group for thrombosis, for which aspirin and/or cytoreductive therapy are recommended [9].

Reports on AI associated with JAK2 V617F-positive ET are extremely rare, with only five documented cases, including ours (Table 5). All reported cases involved male patients older than 60 years. The major clinical symptom was upper abdominal pain. Sever fatigue was noted in two cases, both of which exhibited low cortisol levels. All cases were administered stress dose of hydrocortisone to manage adrenal insufficiency and aspirin for the treatment of ET. Hada *et al.* reported a patient whose platelet count was within the normal range at the onset but subsequently increased to 102 × 10^4^/μl, leading to a diagnosis of ET [5]. This observation underscores the necessity of investigating the JAK2 V617 mutation in cases of unexplained thrombosis, regardless of platelet count.

**Table 5:** Summary of studies reported adrenal hemorrhage (AH)/infarction (AI) caused by JAK2 V617F-positive essential thrombocythemia.

Authors	Sex	Age (years)	Clinical presentation	Serum cortisol levels (μg/dl)	Platelet count at onset (/μl)	CT findings	Patient management		Final steroid dosage
							For adrenal insufficiency	For ET	
Gensous *et al.* [3]	Male	83	(i) epigastric pain (ii) severe fatigue	1.3	34.0×10^4 (*)^	Bilateral adrenal enlargement without contrast enhancement	Stress dose of hydrocortison (NA)	Aspirin ^(***)^ VKA	NA
	Male	82	(i) abdorminal pain (ii) severe fatigue	3.0	52.5×10^4 (**)^	Bilateral adrenal enlargement with high density centrally	Stress dose of hydrocortison (NA)	Aspirin ^(****)^ Hydroxyurea VKA	NA
Iemura *et al.* [4]	Male	64	(i) epigastric pain (ii) left anterior chest pain	3.8	121.7×10^4^	Bilateral adrenal enlargement and inflamamtion of the adjacent tissue	Stress dose of hydrocortison (90 mg)	Aspirin ^(*****)^ Hydroxyurea	NA
Hada *et al.* [5]	Male	76	(i) upper abdominal pain	16.2	24.2×10^4^	Bilateral adrenal enlargement	Stress dose of hydrocortison (NA)	Aspirin (100 mg) Hydroxyurea	10 mg of hydrocortisone
Present case	Male	69	(i) right abdominal pain (ii) left-sided abdominal pain	29.9	54.7×10^4^	Bilateral adrenal enlargement and inflamamtion of the adjacent tissue	Stress dose of hydrocortison (100 mg)	Aspirin (100 mg)	No administration

VKA: vitamin K antagonists, NA: not available.

(*) He was diagnosed with ET 5 months prior to AH onset and treated with hydroxyurea 500 mg/day.

(**) He was diagnosed with ET 10 years prior to AH onset. After 10 years of treatment with hydroxyurea, he was switched to ruxolitinib, which was also ineffective and was temporarily stopped for a few months.

(***) Prior to the onset of AH, aspirin was used for peripheral artery disese and rivaroxaban for atrial fibrillation but was switched to VKA.

(****) Prior to the onset of AH, apixaban was used for atrial fibrillation but was switched to VKA. Hydroxyurea was resumed.

(*****) Prior to the onset of AI, aspirin was used for angina pectoris.

Although the mechanism underlying AI remains poorly defined, the anatomical features of adrenal vasculature may contribute to its development. The adrenal glands receive blood inflow from three adrenal arteries and outflow from only one adrenal vein. This unique structure makes them vulnerable to ischemic events [10]. Fortunately, our patient experienced a favorable clinical course. The administration of aspirin during the early stages of adrenal gland injury may have facilitated this positive outcome.

## Conclusion

We experienced a case of AI as the first thrombotic complication of ET. In patients presenting with abdominal pain, AI is an infrequent but important differential diagnosis. Although rare, it is possible that AI may be caused by ET, so it is important to investigate this disease in the setting of coexisting platelet hyperplasia. Early diagnosis and treatment of the causative disease is also extremely important from the perspective of preventing recurrence of AI.

## Consent

Written informed consent was obtained.

## Guarantor

Yuji Hataya.
